# Case of Sophia Bartlet, Who Was Supposed to Have Undergone Small-Pox a Second Time

**Published:** 1812-02

**Authors:** 


*For the Medical and Physical Journal.*

CASE *of* SOPHIA BARTLET, *who was supposed to have undergone* SMALL-POX *a second Time.*

S. BARTLET, a married woman, aged 31 years, residing at No. 9, Baldwin's-gardens, Gray's-inn-lane, was believed to have had the small-pox when five years old, and there are pits, evidently from that disease, especially on her face. She has borne seven children: six of them had the small-pox, whom she nursed in that disease; and she has lived in chambers where other children have died of it. She remembers having the chicken-pock about a year after the small-pox.

Saturday, the 20th of October, 1811, she buried a child who died in the small-pox, which she suckled. It had been ill a fortnight before the eruption appeared, but was considered as in the process of teething, till three days before the appearance of the eruption, when sickness at stomach and other symptoms occurred. The eruption came out on Sunday, the 6th of October, and the child died on the 17th. Mr. Andrews, of Hatton-garden, attended the child, and informed Dr. Pearson there was no doubt of the disease being small-pox.

Sophia Bartlet was very well herself for a week after the eruption broke out on the child; but was ill two or three days before it died. She was sick at stomach, lost the use of her limbs, and had considerable vertigo. She describes more particularly, that she had no head-ach previously to the eruption; that she was siezed very suddenly with swimming of the head, and, in a great measure, deprivation of the senses: she was also thirsty, hot, and feverish, lost all relish for for food, but it is not certain that she had any rigors. It appears that she was seized with these symptoms two days before the child's death; that she was still worse on Wednesday, Thursday, and Friday, till Saturday, when she was relieved by the coming out of the eruption.

The eruption, perhaps, appeared first on Friday, that is, the day after the child died, or at least part of her face was unusually hot and red on that day.

On Saturday the eruption first distinctly appeared on the right cheek, in the mouth, on the nipples and areola of her breasts; but only here and there on the trunk and lower limbs, and a few on the left cheek.

It appears that the inflammation of the face, which was succeeded by the eruptions, began as early as Tuesday, along with the constitutional affection; as at that time she felt a burning heat, or scalding as she called it, of that part. On Saturday the burning or scalding of the right cheek abated, as well as the constitutional symptoms, but the eruptions grew larger and larger, with less and less fever; and it is probable that they suppurated in four or five days from their first appearance,—that is, by Wednesday or Thursday the 23d or 24th of October. She considers the most severe days of her illness to be Friday and the early part of Saturday; the day before and the day on which the eruptions appeared most distinctly. The child sucked both breasts till two days before its death.

When her eruptions first came out, they were much larger than the child's, and more red at bottom. After Monday they began immediately to suppurate. The child's were much longer in coming to suppuration; and she recollects, that, before those on the child suppurated, they were like water, but that her's were never watery, but contained a purulent fluid from the first, accompanied with great heat; and the left cheek was so swollen as nearly to close the eye. The nipples also swelled, and the pustules were full of yellow matter, which turned black on Sunday the 27th.

There are now (Tuesday, October 29th,) seventeen scabbed eruptions on the right cheek, four on the chin, one on the left temple, one on the lower right arm, one on the lower left arm, and several on both nipples.

She states that there were eruptions on the mouth and tongue, which she attributes to using the spoon which had been in the child's mouth. She attended Mr. Brookes's lecture-room on Friday the 25th, and Dr. Pearson was informed there was then matter in many of the eruptions; but the scabbing process was much advanced, and completely finished by Monday the 28th, when she attended Dr. Pearson's lecturelecture-room. At this time the scabs were not unlike those of the small-pox, but rather more defined. She appears to have had no secondary fever.

On Tuesday the 29th, Sophia Bartlet attended the Vaccine Institution in Broad-street, where the above statement was taken.

On inquiring into the circumstances of this woman's exposure to her child, it appears that the child's face and head, covered with the confluent small-pox, were almost constantly applied to the right side of the face of the mother, and seldom to the left. That the child had many eruptions on the lips, and probably in the mouth, which must necessarily have been applied to the nipples and breasts of the mother. The eruptions in the mouth of the mother are also accounted for, from frequently tasting the food in the spoon, after it had been put into the mouth of the child.

Dr. Pearson, whose inquiries into the circumstances of this case were particularly minute, does not consider it as affording an example, as hath been generally asserted, of small-pox occurring a second time in one who had decidedly already undergone that disease. He does not pretend, however, that the evidence is perfectly complete, because a reliance was necessarily placed upon the statement given by the woman, of the symptoms and progress of the complaint for at least twelve days before he saw her: but still he thinks there are facts enow sufficiently authenticated to warrant his conclusion.

In the first place, there is one circumstance which seems alone decisive, viz. that the eruptions were confined to two parts, the right cheek and the nipples of the two breasts, where they appeared in clusters: and it is obvious that to these parts the virus of the infant was applied in sucking, and lying with its face covered with confluent small-pox upon that cheek. It is true that a pustule also appeared upon the left temple, one on the right, and another upon the left, arm, which may be easily accounted for from an accidental contact of matter with these parts. According also to the woman's account, some eruptions broke out in her mouth, which may be accounted for from matter being introduced by tasting the child's food in the spoon.

2dly, It is not quite clear whether the illness which preceded the eruptions was a fever occasioned by variolous matter, or from the distressed state of mind and fatigues of body from the illness of the infant. But, from the kind and violence of the symptoms, Dr. Pearson thinks it most probable that the fever was excited by the stimulus of the small-pox virus.

3dly, If the history given by the patient can be depended on, the eruption did not proceed through the regular shapes of of pimple, vesicle, pustule, and scabbing, of the small-pox; but some inflammation of the parts took place even on the day of the attack, previous to the eruption, and which increased very considerably up to the day of the eruption; suppuration took place in three or four days after its appearance, without going through the vesicular stage; and the scabbing stage, which was completed when she attended the Institution, did not appear to have been preceded by the usually figured eruptions of small-pox.*

4thly, There does not appear to have been any fever on the 8th, 9th, or 10th, days, from the first breaking out of the eruption.

5thly, The inflammation of the skin appears to have been attended with much more burning pain, heat, and redness, on the third and fourth days from the eruption breaking out, than ever occurs in the small-pox.

Sophia Bartlet was seen at the Institution on the 29th of October, 1811, by Dr. Pearson, Dr. Domcier, E. Brande, esq. J. Doratt, esq. George Grant, esq. R. Ogle, esq. and W. Russel, esq. who concurred in the opinion that her case was a local affection on the parts only, to which variolous

---

* In our Journal, vol. xxvi. p. 423, we gave a cursory notice of this case as an article of intelligence. It was then generally understood to be a second case of small-pox occurring in the same person. From inquiries which we made on the 25th of October, at Mr. Brooks's, of the woman, and from the appearance of the disease, we were of opinion that this "case presented a curious anomaly in the history of *Variola*; and that, if the fever which accompanied it was the genuine eruptive fever of small-pox, it was obvious that the susceptibility of the system had been so modified by the patient having before passed through the disease, that it was incapable of producing pustules but where the variolous fluid was applied by actual contact." Having seen the eruption in an earlier stage than when submitted to the inspection of Dr. Pearson, we are enabled to say, though it had the general character of variola, it differed in some particulars, not easily explained, but sufficiently obvious to an accustomed eye. It had some analogy to that pustular appearance induced on the skin by the repeated application of some irritating material, especially tartarised antimony: in this instance, perhaps, it may have been occasioned by the repeated application of a morbid fluid, by a similar process, but independent of the specific action of the *variolous fluid*. The ingenious investigation of Dr. Pearson, enables us to correct a mistatement in our former notice. The eruption was preceded by, and accompanied with, fever, but it had not the true character of the eruptive fever of small pox; neither did the eruption, as Dr. Pearson has justly remarked, go through the regular stages of the variolous pustule. We acknowledge ourselves indebted to the liberality of the physicians and surgeons of the *Vaccine Institution in Broad-street*, for the minutes of this interesting case.—Editors.

 matter matter had been applied; was accompanied with an eruption of a different character from small-pox, although there was a general affection of the system.

